# The impact of decentralization of health services at the municipal level on health outcomes: evidence from China

**DOI:** 10.3389/fpubh.2024.1392222

**Published:** 2024-06-07

**Authors:** Feng Cui, Shanshan Wang

**Affiliations:** ^1^School of International Trade and Economics, University of International Business and Economics, Beijing, China; ^2^School of Insurance and Economics, University of International Business and Economics, Beijing, China

**Keywords:** decentralization of health services, prefecture-level city, physical health, depression status, cognitive ability

## Abstract

**Objectives:**

Under the system of health decentralization, there are differences in the level of expenditure decentralization at different levels of government, and their impact on residents’ health may also be different. This paper is one of the first to evaluate the effect of decentralization of health services at the municipal level from a multi-dimensional health perspective.

**Data and methods:**

This paper uses the data of expenditure decentralization of health services at the municipal level to match the panel data from the China Household Panel Survey (CFPS) from 2010 to 2018, and uses the logit model, ordered logit model and two-way fixed effects model to empirically analyze the impact of health decentralization at the municipal level on health outcomes.

**Results:**

Based on the perspective of multi-dimensional health, from the three aspects of physical health, depression status and cognitive ability, the six sub-indicators of self-rated health, BMI standards, depression scores (summation method), depression scores (factor method), phrase test scores and mathematics test scores are discussed separately. The results show that the decentralization of health services at the municipal level has a significant promotion effect on the multi-dimensional health of residents.

**Conclusion:**

The decentralization of health services at the municipal level has important theoretical significance for promoting the reasonable division of medical and health powers and expenditure responsibilities between provincial and municipal governments, improving the efficiency of health expenditure funds, and establishing a fiscal system that matches financial resources.

## 1 Introduction

Health is an inevitable requirement for promoting the all-round development of people and the basic condition for economic and social development. The Healthy China 2030 strategy puts public health in a strategic position of priority development for the first time, and clearly states that the implementation of government policies should promote the improvement of people’s health and achieve universal health coverage, which reflects the country’s great emphasis on health (1). As an important livelihood undertaking to protect the health of the whole people, the development of medical and health care is naturally inseparable from the government’s management of public health affairs and investment in medical and health matters.

During the last two decades, the Chinese government has continuously increased its investment in healthcare system, and the medical and health conditions of its citizens have been greatly improved. Between 2000 and 2018, total health expenditures grew rapidly, with an average annual growth rate of approximately 15.22% (2). After excluding the impact of inflation, the actual average annual growth rate was 12.75% (2, 3). At the same time, the scale of government health expenditure continued to expand, accounting for 27.74% of the total health expenditure from 15.84% (2). However, in the context of increasing population aging, the sustainability of the medical security system faces severe challenges, and the efficiency of the use of health financial funds still needs to be improved. The problem of low efficiency of financial health funds stems from the complex financial management system. The healthcare system in China is very large due to its coverage of a wide range of people. According to the national health expenditure structure (4), the healthcare system mainly includes four aspects: public health, medical security, family planning and capacity building. Each aspect includes a number of health matters, a total of 50 items. Meanwhile, since the Chinese government has a five-level government structure, including the central government, provinces, cities, counties, and towns, these health matters are also listed as common matters or separate matters by governments at all levels, so the actual health matters are more complicated.

The main problems in the division of intergovernmental health fiscal powers and expenditure responsibilities in China can be attributed to three aspects: First, affected by the isomorphism of government agencies, the division of fiscal powers among some governments is unclear and unscientific, and the responsibilities are overlapping; second, due to the influence of political centralization and economic decentralization, higher-level governments tend to shift their powers downwards, causing lower-level governments to bear more expenditure responsibilities; third, affected by the imperfect tax-sharing system and inter-governmental transfer payment system, local fiscal resources are not guaranteed, resulting in a mismatch between financial resources, administrative powers and expenditure responsibilities. In addition to the above-mentioned issues, issues such as population aging and changes in disease spectrum have created a large amount of uncertainty, posing challenges to the sustainability of the healthcare system.

In recent years, defining the relationship between intergovernmental health expenditures is core to the reform of Chinses health system. In August 2018, China issued the “Reform Plan for the Division of Fiscal Powers and Expenditure Responsibilities between Central and Local Governments in the Medical and Health Fields (5).” (called “The Reform Plan between Central and Local Governments”) Through the division of intergovernmental fiscal and health powers and expenditure responsibilities, problems such as “there are various central and local sharing ratios in Chinese medical and health field, and the division of expenditure responsibilities is not reasonable, some projects are scattered and managed by multiple parties, and the use of fiscal funds is not efficient” can be solved. Since then, each province has successively issued reform plans for the division of health rights and expenditure responsibilities between provinces and municipalities on the basis of complying with the reform plan between central and local governments. In this context, exploring the impact of decentralization of health services at the municipal level on health outcomes has theoretical and practical significance.

Decentralization of health services means that higher-level governments delegate part of their fiscal power to local governments, giving them the autonomy to arrange and implement health matters (6). According to the reform plan for the division of fiscal powers and expenditure responsibilities in the health sector in China, the health matters undertaken by governments at all levels actually correspond to the scale of fiscal expenditures (5). Therefore, decentralization of health services in this study refers to both administrative decentralization and fiscal decentralization. In practice, decentralization of health services can be measured through comprehensive indicators, which can not only record the proportion of local government health expenditures in total expenditures in the form of percentages, but also take into account the autonomy of local governments in making health expenditure decisions. In addition, the following literature introduces methods for assessing the effects of decentralization. For example, Barankay & Lockwood (2007) used the proportion of municipal government education expenditures in Switzerland to the sum of municipal and state government expenditures in the region to measure the degree of municipal government education fiscal decentralization (7). A set of panel regressions are also used to assess the productive efficiency of government, which is similar to the approach to health decentralization. Rubio (8) also used the proportion of municipal and provincial governments in total health expenditures when measuring Canada’s decentralization, and used the fixed effects version of the panel data estimator to calculate the health effect. Soto et al. (9) measured the degree of decentralization in Colombia as the proportion of health expenditure controlled by municipal governments to the sum of all municipal health expenditures, then used an unbalanced fixed-effect regression model with robust errors to assess the health effect. However, Faguet and Sánchez (10) and Espasa et al. (11) used a different approach, they introduced dummy variables on this basis to measure whether the municipal government’s spending decisions are independent of local governments, then used the difference-in-difference model to test whether decentralization improves welfare levels.

There are several related international studies on the impact of fiscal decentralization on public health (9, 12, 13), while there are few Chinese studies and the conclusions are inconsistent (14, 15). Furthermore, there is little literature studying the impact of decentralization of health services on residents’ health in China.

Theoretically, the views of fiscal decentralization to improve public health and increase overall welfare can be summarized as the following two: One view is that local governments make public decisions can shorten the distance between policy makers and the grassroots. That is, decentralization can allow people to participate in the formulation and implementation of public policies, so local government public policies are more flexible (16, 17). Another view is that decentralization enables local governments in each jurisdiction to assume more responsibility for health expenditures and have more autonomy in decision-making (18). Decentralization may also lead to intensified competition among local governments, which can improve the supply efficiency of domestic medical and health products. Some studies use infant and child mortality as proxy variables for health outcomes and draw relatively consistent conclusions. That is, fiscal decentralization has significantly reduced infant and child mortality, and this effect is more obvious in low-income areas (19–21). On this basis, scholars further discussed the impact of local fiscal revenue and expenditure structure on this effect. The study believes that fiscal decentralization is likely to improve health outcomes only when local fiscal security capabilities are sufficient. Meanwhile, the proportion of health expenditures and education expenditures in GDP has increased, making the positive effect of fiscal decentralization on health outcome more obvious (8). Limited by the availability of data, the above analysis uses the indicator of fiscal decentralization and does not involve decentralization of health services, therefore, it is still insufficient.

Objectively speaking, the effect of the fiscal decentralization depends on many factors such as politics, institutions, and revenue centralization (22). The decentralization system should include a clear division of responsibilities and a corresponding accountability system. Therefore, the impact of decentralization of health services on residents’ health may also be uncertainty (12, 23). Take a study in Spain as an example, which systematically analyzed the effects of decentralization reform of health services in Spanish in different time periods and found that patient satisfaction with hospital conditions and hospital care did not improve between 1996 and 2009 (24). As for the reasons for low satisfaction with medical conditions, some studies believe that the decentralization reform of health services implemented in Spain since 1981 is more based on political and administrative decentralization considerations than on protecting the health of the subjects (25). On this basis, follow-up research used quasi-natural experiments and difference-in-difference methods to prove that Spain’s decentralization reform of health services improved residents’ health (23). In addition, for developing countries, health decentralization reforms may aggravate the uneven degree of medical and health conditions among regions (26), which will have a negative impact on the improvement of residents’ health (27).

Compared with international studies (9, 12, 13), there are only a few studies based on Chinese data (14, 28). The following two papers are worthy of attention, and there is obvious controversy over the impact of fiscal decentralization on health outcomes. First, Uchimura and Jütting (28) used data from 26 provinces in China from 1995 to 2001 to empirically test the impact of fiscal decentralization at provincial level on infant mortality and found that fiscal decentralization at provincial level can reduce infant mortality. Meanwhile, when health decentralization at the county level (the proportion of health expenditures of county-level government in health expenditures of provincial government) remains unchanged, transfer payments from provincial governments to county-level governments can also improve the health outcome of county-level regions. Second, Peng et al. (14) draw different conclusions by analyzing the data of 30 provinces in China from 1995 to 2009. The results found that both revenue decentralization and expenditure decentralization at the provincial level are not conducive to health outcome (population mortality), while transfer payments from the central government to provincial governments can improve provincial health outcome to a certain extent.

Judging from the practice of fiscal decentralization reform, the main reasons why fiscal decentralization reform is easily questioned are as follows: First, for medical and health services with strong externalities, decentralization reforms of health services are not conducive to achieving economies of scale. Instead, it would be more efficient for higher-level governments to provide such medical services nationwide. Second, decentralization means that local governments have to bear more administrative responsibilities, while local governments are weaker than higher-level governments in terms of staffing and administrative agency setup, which increases the administrative pressure on local governments. Third, the policy goals of local public health are not necessarily consistent with the actual medical needs of the people. Due to various challenges in the healthcare sector and the continuous increase in health expenditure pressure (29), policy makers may prioritize achieving other policy goals, resulting in decentralization of health services not being able to meet the diverse medical needs of the public (23).

It is notable that when analyzing the impact of decentralization of health services on residents’ health, local governments should not be generally regarded as a homogeneous whole. Under the decentralization system of health services, there are differences in the level of expenditure decentralization at different levels of government, and their impact on residents’ health may also be different. Meanwhile, resident health encompasses multiple aspects, such as physical health, depression status and cognitive ability. Based on this, the purpose of this study is to assess the effect of decentralization of health services at the municipal level on residents’ multi-dimensional health in China. In addition, this study is the first to take decentralization of health services at the municipal level as the research object, and matches it with CFPS data to empirically analyze the impact of decentralization of health services on public health in China, which not only enriches relevant research on the welfare effects of decentralization of health services, and also provide ideas for scientifically defining the distribution relationship of intergovernmental fiscal health expenditures and achieving universal health coverage.

## 2 Materials and methods

### 2.1 Data source

The China Family Panel Survey (CFPS) collects data at three levels: individual, family and community. The survey subjects include a total of 16,000 urban and rural households, which overall reflects the changes in China’s economy, society, education and health. The project began test surveys in 2008 and 2009, and then carried out formal follow-up surveys in 2010, 2012, 2014, 2016, 2018 and 2020. This paper uses micro data of formal surveys from 2010 to 2018 to match health decentralization data, and conducts an empirical study on the impact of decentralization of health services at the municipal level on residents’ health. The reason for choosing this time window as the sample is that considering the impact of the COVID-19 epidemic, the health decentralization indicators at the municipal level after 2019 have changed significantly compared with previous years, so the merged data set does not include 2020 and later year. Among them, residents’ health indicators, personal characteristics and family characteristics variables come from CFPS data; the health expenditure data of provincial government come from the “China Fiscal Yearbook” over the years; The health expenditure data of prefecture-level city come from financial budget and final account reports, national economic and social development statistical bulletins, and municipal statistical yearbook that were manually collected and compiled from 2010 to 2018; Other prefecture-level variables come from the “China Urban Statistical Yearbook” and “China Urban Construction Statistical Yearbook.”

### 2.2 Variables

#### 2.2.1 Explained variables and core explanatory variables

The explained variable is the residents’ health, which is discussed from the three aspects of physical health, depression status and cognitive ability, and measured by six sub-indicators of self-rated health, BMI standards, depression scores (summation method), depression scores (factor method), phrase test scores and mathematics test scores. Among them, self-rated health is a commonly used indicator in health economics, which can better reflect the overall health status of an individual. However, as a subjective indicator, its measurement is easily affected by individual heterogeneous characteristics, and therefore it is easy to form measurement errors (30, 31). Self-rated health comes from the questionnaire “How do you think your health is?” Referring to the approach of (32–34), “very Healthy,” “very healthy” and “relatively healthy” are combined into “self-rated health is good” and assigned a value of 1; “average” and “unhealthy” are combined into “self-rated health is poor” and assigned a value of 0. BMI standards is based on the individual’s height and weight indicators to calculate the BMI index (35), that is, BMI index = weight (kg)÷height (m)^2, and then according to the evaluation standards of the “Guidelines for the Prevention and Control of Overweight and Obesity in Adults in China (2021)” (36), which regulates that BMI < 18.5 is underweight, 18.5 ≤ BMI < 24 is healthy weight, 24.0 ≤ BMI < 28 is overweight, and BMI ≥ 28 for obesity, then assigning obesity a value of 1, underweight and overweight a value of 2, and healthy weight a value of 3. The depression degree indicator variable comes from the behavioral and mental state part of the CFPS (34), which is calculated based on the respondents’ responses to “feeling depressed, unable to cheer up when doing anything,” “nervous,” “difficulty staying calm,” “no hope for the future,” “difficulty doing anything,” and “life is meaningless.” The phrase test and mathematics test come from the cognitive module of CFPS (33). The higher the score, the stronger the cognitive ability of the individual.

In addition, this study also selects some alternative indicators related to residents’ health for robustness testing (35). Among them, physical discomfort is measured by the question “Have you had physical discomfort in the past two weeks” in questionnaire. If yes, it is assigned a value of 1, otherwise it is assigned a value of 0. Self-assessed memory comes from the behavioral and mental state module in the questionnaire, that is, “What proportion of you can remember the main things that happened to you in a week?” According to the answers “can only remember a little bit,” “Can only remember a few,” “can remember half,” “can remember most,” and “can remember completely,” are assigned values from 1 to 5, respectively. The variable of daily activity ability comes from the physical test part in the questionnaire, including whether to independently perform or use “outdoor activities,” “eating,” “kitchen activities,” “public transportation,” “shopping,” “cleaning and hygiene” and “laundry,” and the score is assigned according to the number of items that residents can complete independently.

The core explanatory variable is the decentralization of health services at the municipal level, referring to the practice of (37–40), the expression is “decentralization of health services at the municipal level = final accounts of health expenditures of municipal government / final accounts of health expenditures of the whole province.” This indicator measures the distribution of fiscal health expenditures between provincial and municipal governments from the perspective of expenditure decentralization, and reflects to a large extent the power and expenditure responsibilities of municipal governments in the medical and health field.

#### 2.2.2 Control variables

Referring to relevant literature, control variables are divided into personal characteristics variables, family characteristics variables, and prefecture-level city characteristics variables (34). Among them, personal characteristics variables include: (a) gender; (b) age; (c) household registration type; (d) marital status; (e) years of education; (f) work status, reflecting whether the individual has a job; (g) political outlook, reflecting whether the individual is a party member; (h) medical insurance, reflecting whether the individual has medical insurance. Family characteristics variables include: (a) Family *per capita* income; (b) Household financial assets, reflecting the investment scale of family risk assets; (c) Family entertainment expenditure, reflecting family consumption for leisure and entertainment. Prefecture-level city characteristic variables include: (a) Secondary industry, measured by “the proportion of secondary added value in GDP,” reflects the changes in the regional industrial structure; (b) Openness, reflecting the proportion of total export–import volume to regional GDP; (c) Rate of GDP growth, reflecting the annual economic development of the region. The remaining variables employed in the analysis are described in Table 1.

**Table 1:** Definition of the variables used in the study.

Variable		Definition (unit)	Mean	SD	Min	Max
Explained variables:						
Main health indicators	Physical health	Self-rated health: “very Healthy,” “very healthy” and “relatively healthy” =1; “average” and “unhealthy” = 0	0.7106	0.4535	0	1
		BMI standards: obesity = 1; underweight and overweight = 2; healthy weight = 3	2.1300	0.8437	1	3
	Depression status	Summation method: calculate the depression status of the respondents based on the questionnaire	26.9332	3.8774	6	30
		factor method: calculate the depression status of the respondents based on the questionnaire	−0.00003	1.0000	−5.4570	0.7797
	Cognitive ability	phrase test score: calculated based on the Guttman scale	17.8513	10.7665	0	34
		mathematics test score: calculated based on the Guttman scale	9.7040	6.4964	0	24
Other health indicators	feel unwell	whether you feel unwell in the past 2 weeks	0.2934	0.4553	0	1
	Self-evaluation memory	To what extent you can remember the last week	2.6587	1.3225	1	5
	activity of daily living	How many daily activities can be completed independently	6.6615	1.0577	0	7
Core explanatory variable:						
Decentralization of health services at the municipal level		Final account of health expenditure of municipal government / final account of health expenditure of provincial government (%)	7.9245	4.5098	0.0002	41.1960
Robustness test variable:						
decentralization of health services at the municipal level		*Per capita* municipal health expenditure / (*per capita* municipal health expenditure + *per capita* provincial health expenditure + *per capita* central health expenditure)	0.9087	0.0781	0.4819	0.9801
Control variable:						
Individual characteristic variables:						
Gender		Male = 1; female = 0	0.4960	0.5000	0	1
Age		Respondent’s age	45.1211	17.6195	9	110
Household registration type		Rural area = 1; urban area = 0	0.6964	0.4598	0	1
Marital status		Married = 1; other = 0	0.7016	0.4576	0	1
Education		Actual years of education received	7.3102	4.8684	0	23
Work		Having a job = 1; No work = 0	0.5821	0.4932	0	1
Political outlook		Communist Party member = 1; other = 0	0.0590	0.2356	0	1
Medical insurance		Participate in medical insurance = 1; other = 0	0.7894	0.4077	0	1
Family characteristic variables:						
Family income		Total household income (in thousands of yuan)/number of family members	17.4381	46.4560	0	5,660
Financial assets		Scale of financial assets such as stocks and funds (in thousands of yuan)	45.7925	189.745	0	10,000
Entertainment expenditure		expenditures for cultural and entertainment in the past year (in thousands of yuan)	5.2004	11.1124	0	630
City characteristic variables:						
Secondary industry		The proportion of secondary added value in GDP	46.3000	10.0951	12.1900	89.7500
Openness		The proportion of total export–import volume to regional GDP	1.8689	1.9648	0.0012	19.7828
GDP growth		(GDP of the current year - GDP of the previous year) / GDP of the previous year	9.4932	5.9019	−12.300	109.000
Instrumental variable:						
Real estate investment		Investment scale of real estate in the region (100 million yuan)	214.065	322.5122	0.2934	2111.595

### 2.3 Empirical methodology

On the basis of the data form of residents’ health, we can derive the following regression equation to analyze the relationship between decentralization of health services at the municipal level and residents’ multi-dimensional health.

#### 2.3.1 Baseline regression model

##### 2.3.1.1 Logit model

According to the discrete characteristics of the explained variables, this study selects the logit model for regression. The model typically uses maximum likelihood estimation, a widely accepted statistical method, to determine parameters. In addition, the regression results of the model are relatively stable, and the prediction results of the model are relatively consistent even on different samples. Referring to the practice of (41, 42), this paper constructs the following panel Logit model:


(1) $$\;P\left(Y_{it}=1|X_{it},\beta ,u_{i}\right)=\Lambda \left(u_{i}+X_{it}^{\prime }\beta \right)=\frac{e^{u_{i}+X_{it}^{\prime }\beta }}{1+e^{u_{i}+X_{it}^{\prime }\beta }}$$

In Equation (1), $\Lambda \left(.\right)$ is the cumulative distribution function of the logistic distribution, and the explained variable is the self-rated health; $X_{it}\;$ is the explanatory variable vector, including decentralization of health services at the municipal level, personal characteristic variables, family characteristic variables, and city characteristic variables; β is the coefficient vector, and β is the individual effect.

##### 2.3.1.2 Ordered logit model

The Ordered Logit model is used to process ordered categorical result variables, that is, there is a certain order relationship between the categories of the explained variables. The Ordered Logit model provides a variety of statistical tests and graphical tools to evaluate the fitting of the model, such as the Brant test, which helps identify and correct problems in the model. Therefore, the model is widely used in the analysis of ordered classified data in social sciences, health sciences, educational research and other fields. According to the ordered and discrete characteristics of the BMI standards, this paper matches the Ordered Logit model for estimation (43). The specific econometric equation setting form is as follows:


(2) $$\;Y_{i}=F\left(\beta_{0}+\beta_{1}Con\_Fd+X_{i}^{\prime }\varphi +\varepsilon_{i}\right)$$

(3) $$\;Y_{i}^{*}=\beta_{0}+\beta_{1}Con\_Fd+X_{i}^{\prime }\varphi +\varepsilon_{i}$$

(4) $$\;F\left(Y_{i}^{*}\right)=\{\begin{array}{ccc} 1, & if & Y_{i}^{*}<\delta_{1}\\ 2, & if & \delta_{1}<Y_{i}^{*}<\delta_{2}\\ 3, & if & \delta_{2}<Y_{i}^{*}<\delta_{3}\\ \vdots & & \\ j, & if & Y_{i}^{*}>\delta_{j-1} \end{array}$$

If the disturbance term satisfies the conditions of the standard normal distribution, which is $\varepsilon_{i}\sim N(0,\sigma^{2}$ ), then the probability of $Y_{i}\;$ taking the value *j* is as follows:


$$\;P_{ij}=P\left(Y_{i}=j\right)$$

(5) $$=\Phi \left(\delta_{j}-\beta_{0}-\beta_{1}Con\_Fd_{i}-X_{i}^{\prime }\varphi \right)\;$$

$$-\Phi \left(\delta_{j-1}-\beta_{0}-\beta_{1}Con\_Fd_{i}-X_{i}^{\prime }\varphi \right)$$

In Equations (2–5), the subscript *i* represents the i-th individual; Con_Fd is the core explanatory variable, representing the decentralization of health services at the municipal level; φ represents its corresponding coefficient vector; $Y_{i}^{*}$ is unobservable latent variables, its form satisfies the equation of (3), and its selection rule satisfies the equation of (4); $\delta_{1}<\delta_{2}<\delta_{3}<\ldots <\delta_{J-1}$ represents the parameter to be estimated and is also called the cut point.

##### 2.3.1.3 Two-way fixed effects model

According to the characteristic that the depression status and cognitive ability are continuous variables, this paper selects the two-way fixed effects model as the benchmark model (7–9). The model can control individual-specific effects that do not change with time and time-specific effects that do not change with individuals, thereby reducing the impact of omitted variable bias and measurement error. In addition, when there is inter-individual and temporal non-observed heterogeneity, the model can accurately estimate the impact of explanatory variables on the explained variable. The specific regression equation is set as follows:


(6) $$\begin{array}{c} Healthihct=\alpha +\beta 1Con\_Fdct+Xict\;\theta \\ +Niht+Εct\delta +\mu i+\lambda t+\varepsilon ihct \end{array}$$

Among them, the subscripts *i*, *h*, *c*, and *t* represent individuals, families, cities, and years respectively; *Health_ihct_* represents the health level of individual *i* in year *t*, such as depression scores (summation method), depression scores (factor method), phrase test scores and mathematics test scores; the core explanatory variable *Con_Fd_ct_* represents the decentralization of health services of prefecture-level city *c* in year *t*; *X_ict_*、*N_iht_* and 𝐸*_ct_*, respectively represent a series of vectors of individual-level, family-level and city-level variables to control individual characteristics, family characteristics and city characteristics; *μ_i_* represents individual fixed effects; *λ_t_* represents year fixed effects; *ε_ihct_* represents the random disturbance term. This paper focuses on *β*_1_ in Equation (6), which represents the impact of decentralization of health services of prefecture-level city on residents’ health after controlling for characteristic variables at the individual level, household level, and prefecture-level city level.

#### 2.3.2 Instrumental variable method

Generally speaking, due to reasons such as omitted variable bias, measurement error, and reverse causality, the impact of decentralization of health services at the municipal level on residents’ health may have endogeneity problems. In order to avoid the above problems causing bias to the estimation results, referring to the practice of (44), this paper uses the “completed amount of real estate investment” as the instrumental variables of decentralization of health services to enhance the credibility of the research conclusions. There are two reasons for using this indicator as an instrumental variable: First, decentralization of health services at the municipal level measures the proportion of municipal health expenditures to the provincial health expenditures. Changes in the proportion of health expenditures of municipal government require corresponding financial resources as a guarantee, and the completed amount of real estate investment can reflect the local land finance situation to a large extent, so it is relevant. Second, the completed amount of real estate investment has no direct correlation with the omitted variables and residents’ health, which satisfies exogeneity. The regression equation of the two-stage least squares method (2SLS) is shown in Equations (7, 8). The first stage represents the regression of instrumental variables on decentralization of health services at the municipal level, and the second stage represents the regression of the fitted value of decentralization of health services obtained from the first stage on the residents’ health. The meaning of other variables is consistent with the baseline regression equation. This paper focuses on the coefficient *β*.


(7) $$\begin{array}{c} Two\;stage:Healthihct=\alpha +\beta \hat{Con\_F}d\mathrm{ct}+Xict\;\theta \\ +Niht+Εct\;\delta +\mu i+\lambda t+\varepsilon ihct \end{array}$$

(8) $$\begin{array}{l} Firststage:Con\_Fdct=\omega +\sigma Inv\_houct+Xict\;\theta \\ +Niht+Εct\;\delta +\mu i+\lambda t+\varepsilon ihct \end{array}$$

## 3 Results

### 3.1 Baseline regression results

Table 2 reports the baseline regression results on the impact of decentralization of health services at the municipal level on residents’ health. Estimation results show that decentralization of health services at the municipal level can improve residents’ physical health, alleviate depression, and improve cognitive abilities. In terms of physical health, this paper uses self-rated health and BMI standards for measurement. Among them, the logit model regression results show that decentralization of health services at the municipal level has a positive impact on residents’ self-rated health, and it is significant at the 1% level. The regression results of the ordered logit model show that decentralization of health services at the municipal level has a positive impact on residents’ BMI standards, and is significant at the 5% level. In terms of depression status, this paper uses the summation method and factor method to measure individual depression status. The regression results of the two-way fixed effects model show that the decentralization of health services at the municipal level can reduce residents’ depression and is beneficial to residents’ mental health, and both are significant at the 1% level. In terms of cognitive ability, this paper uses individual phrase test scores and mathematics test scores to measure it. The two-way fixed effects regression results show that the decentralization of health services at the municipal level is conducive to improving individuals’ phrase test and mathematics test scores, and they are significantly positive at the 1 and 5% levels, respectively. Therefore, the overall regression results of this paper support the theoretical inference that decentralization of health services at the municipal level can improve residents’ health.

**Table 2:** The impact of decentralization of health services on residents’ health.

Variables	Physical health		Depression status		Cognitive ability	
	Self-rated health	BMI standards	Summation method	Factor method	Self-rated health	BMI standards
	(1)	(2)	(3)	(4)	(5)	(6)
Decentralization of health services	0.0059***	0.0031**	−0.0406***	−0.0106***	0.0281***	0.0086**
	(0.0018)	(0.0015)	(0.0058)	(0.0015)	(0.0073)	(0.0037)
Gender	0.3015***	0.0351***	0.4089***	0.1054***	1.2170***	0.3874***
	(0.0164)	(0.0134)	(0.0514)	(0.0133)	(0.0689)	(0.0355)
Age	−0.0376***	−0.0132***	−0.0117***	−0.0032***	−0.1387***	−0.0750***
	(0.0006)	(0.0005)	(0.0020)	(0.0005)	(0.0024)	(0.0012)
Household registration type	0.0052	0.2845***	0.0551	0.0163	−0.9245***	−0.2819***
	(0.0205)	(0.0165)	(0.0747)	(0.0193)	(0.0874)	(0.0466)
Marital status	−0.2036***	0.1232***	0.6332***	0.1649***	0.2342***	−0.6724***
	(0.0201)	(0.0174)	(0.0725)	(0.0187)	(0.0886)	(0.0459)
Education	0.0566***	0.0326***	0.0825***	0.0213***	1.4131***	0.9672***
	(0.0021)	(0.0017)	(0.0072)	(0.0018)	(0.0091)	(0.0048)
Work	0.1777***	−0.1323***	0.0883*	0.0232*	−0.7011***	−0.1896***
	(0.0173)	(0.0147)	(0.0528)	(0.0136)	(0.0745)	(0.0384)
Political outlook	0.1887***	−0.1070***	0.3913***	0.1007***	1.7253***	1.2800***
	(0.0355)	(0.0272)	(0.0894)	(0.0231)	(0.1639)	(0.0773)
Medical insurance	−0.0028	0.2022***	0.2751***	0.0710***	−0.3144***	−0.3866***
	(0.0229)	(0.0179)	(0.0613)	(0.0158)	(0.0859)	(0.0453)
Family income	0.0022***	−0.0001	0.0110***	0.0029***	0.0049***	−0.0000
	(0.0006)	(0.0002)	(0.0031)	(0.0008)	(0.0016)	(0.0006)
Financial assets	0.0002**	−0.0000	−0.0001	−0.0000	−0.0001	−0.0001
	(0.0001)	(0.0000)	(0.0011)	(0.0003)	(0.0002)	(0.0001)
Entertainment expenditure	0.0014	0.0036***	0.0050*	0.0013*	0.0186***	0.0236***
	(0.0009)	(0.0008)	(0.0026)	(0.0007)	(0.0036)	(0.0035)
Secondary industry	0.0086***	0.0004	0.0089***	0.0021***	0.0287***	0.0182***
	(0.0010)	(0.0008)	(0.0029)	(0.0008)	(0.0041)	(0.0021)
Openness	0.0035	−0.0038	0.1034***	0.0263***	0.0135	0.0190***
	(0.0043)	(0.0032)	(0.0119)	(0.0031)	(0.0146)	(0.0072)
GDP growth	0.0017	0.0057***	−0.0729***	−0.0186***	−0.1454***	−0.0497***
	(0.0014)	(0.0012)	(0.0122)	(0.0032)	(0.0177)	(0.0093)
Individual fixed effects	Y	Y	Y	Y	Y	Y
Time fixed effect	Y	Y	Y	Y	Y	Y
*N*	100,677	100,677	22,930	22,930	38,024	38,024
Pseudo R^2^ / R^2^	0.1536	0.1934	0.0377	0.0380	0.6174	0.7218

(1) This paper uses the logit model to estimate self-rated health, the ordered logit model to estimate BMI standards, and the two-way fixed effects model to estimate depression status and cognitive ability; (2) Brackets indicates robust standard errors; ***, **, and * indicate results are at the 1, 5, and 10% significance levels, respectively. The following tables in this study are the same.

### 3.2 Explanatory variables are lagged by one period

Since it is difficult to construct strictly exogenous instrumental variables for fiscal decentralization variable in panel data, some literature selects the lag period of the fiscal decentralization variable as the instrumental variable. Referring to existing literature (39, 45, 46), this paper selects the lag period of decentralization of health services at the municipal level as an instrumental variable, and robustness testing is performed. The regression results in Table 3 show that decentralization of health services at the municipal level has a significant positive impact on self-rated health and BMI standards; Decentralization of health services at the municipal level has a significant negative impact on depression status (summation method) and depression status (factor method); Decentralization of health services at the municipal level has a significant positive impact on the phrase test and mathematics test. This verifies the basic assertion that decentralization of health services at the municipal level is conducive to improving the residents’ health.

**Table 3:** Endogeneity discussion—explanatory variables are lagged by one period.

Variables	Physical health		Depression status		Cognitive ability	
	Self-rated health	BMI standards	Summation method	Factor method	Self-rated health	BMI standards
	(1)	(2)	(3)	(4)	(5)	(6)
Decentralization of health services	0.0055***	0.0029**	−0.0367***	−0.0096***	0.0268***	0.0084**
	(0.0017)	(0.0014)	(0.0056)	(0.0014)	(0.0072)	(0.0036)
Individual characteristics	Y	Y	Y	Y	Y	Y
Household characteristics	Y	Y	Y	Y	Y	Y
City characteristics	Y	Y	Y	Y	Y	Y
Individual fixed effects	Y	Y	Y	Y	Y	Y
Time fixed effect	Y	Y	Y	Y	Y	Y
*N*	107,791	107,791	22,930	22,930	38,132	38,132
Pseudo R^2^ / R^2^	0.1514	0.1802	0.0375	0.0378	0.6173	0.7216

### 3.3 Instrumental variable method

Referring to relevant literature (44), This paper selects the completed amount of real estate investment as instrumental variables for decentralization of health services at the municipal level, then uses the two-stage least squares method for estimation. The estimation results are shown in Table 4. In the first stage regression, the regression coefficients of the completed amount of real estate investment are all significantly positive at the 1% level, indicating that the higher the completed amount of real estate investment in region, the higher the level of health decentralization at the municipal level. There is a significant positive relationship between them. This meets the correlation requirement of instrumental variables. Meanwhile, the selected instrumental variables passed the identification deficiency test (Kleibergen-Paap rk LM statistic) and the weak instrumental variable test (Cragg-Donald Wald F statistic), indicating that the instrumental variables are effective. The second-stage regression results show that decentralization of health services at the municipal level can improve residents’ physical health, reduce depression, and improve cognitive abilities. After considering potential endogeneity issues in model, the regression results still support basic assertion that decentralization of health services at the municipal level is conducive to improving the residents’ health.

**Table 4:** Endogeneity discussion—two-stage least squares method (2SLS).

Estimation of second stage						
Variables	Physical health		Depression status		Cognitive ability	
	Self-rated health	BMI standards	Summation method	Factor method	Self-rated health	BMI standards
	(1)	(2)	(3)	(4)	(5)	(6)
Decentralization of health services	0.0033*** (0.0005)	0.0022*** (0.0008)	−0.0422*** (0.0147)	−0.0110*** (0.0038)	0.0542*** (0.0133)	0.0266*** (0.0068)
Individual characteristics	Y	Y	Y	Y	Y	Y
Household characteristics	Y	Y	Y	Y	Y	Y
City characteristics	Y	Y	Y	Y	Y	Y
Individual fixed effects	Y	Y	Y	Y	Y	Y
Time fixed effect	Y	Y	Y	Y	Y	Y
*R* ^2^	0.1668	0.3568	0.0399	0.0402	0.6183	0.7249
Estimation of first stage						
Real estate investment	0.0088^***^ (0.0000)	0.0088^***^ (0.0000)	0.0127*** (0.0001)	0.0127*** (0.0001)	0.0097*** (0.0001)	0.0097*** (0.0001)
Kleibergen-Paap rk LM statistic	1.4e+04	1.4e+04	2406.10	2406.10	4833.70	4833.70
(*p* value)	0.0000	0.0000	0.0000	0.0000	0.0000	0.0000
Cragg-Donald Wald F statistic	5.3e+04	5.3e+04	7032.70	7032.70	1.9e+04	1.9e+04
Critical value of Stock-Yogo	16.38	16.38	16.38	16.38	16.38	16.38
*N*	97,906	97,906	22,930	22,930	35,781	35,781

### 3.4 Replacing the measurement method of health

Referring to the research ideas of multidimensional health of existing literatures (35), this paper replaces the measurement method of individual health, using “whether you feel unwell in the past two weeks,” “to what extent you can remember the last week,” and “how many daily activities can be completed independently” to measure the individual’s health and conduct robustness testing. The regression results in Table 5 show that decentralization of health services at the municipal level can reduce residents’ physical discomfort, improve short-term memory ability, and enhance daily activities. In other words, decentralization of health services at the municipal level can generally improve the health level of residents. Therefore, the regression results of replacing the health measurement method are basically consistent with the baseline regression results.

**Table 5:** Robustness test—replacing the measurement method of health.

Variables	Feel unwell		Self-evaluation memory		Activity of daily living	
	(1)	(2)	(3)	(4)	(5)	(6)
Decentralization of health services	−0.0009**	−0.0007*	0.0037***	0.0036***	0.0033**	0.0032*
	(0.0003)	(0.0003)	(0.0009)	(0.0009)	(0.0017)	(0.0017)
Individual characteristics	Y	Y	Y	Y	Y	Y
Household characteristics	Y	Y	Y	Y	Y	Y
City characteristics	Y	Y	Y	Y	Y	Y
Individual fixed effects	N	Y	N	Y	N	Y
Time fixed effect	Y	Y	Y	Y	Y	Y
*N*	106,397	106,397	93,716	93,716	18,488	18,488
Pseudo R^2^ / R^2^	0.0483	0.0501	0.0959	0.0962	0.1048	0.1048

### 3.5 Changing the measurement method of health decentralization

The indicator of decentralization of health services at the municipal level in this paper is represented by “decentralization of health services at the municipal level = final account of health expenditure of municipal government / final account of health expenditure of provincial government.” To ensure the robustness of regression results, refer to (47, 48), for the construction method of decentralization of health services. This paper adopts “decentralization of health services at the municipal level = *per capita* municipal health expenditure / (*per capita* municipal health expenditure + *per capita* provincial health expenditure + *per capita* central health expenditure)” to measure. The estimation results in Table 6 show decentralization of health services at the municipal level is still conducive to improving residents’ physical health, mental health, and cognitive abilities after changing the measurement method of health decentralization.

**Table 6:** Robustness test—changing the measurement method of health decentralization.

Variables	Physical health		Depression status		Cognitive ability	
	Self-rated health	BMI standards	Summation method	Factor method	Self-rated health	BMI standards
	(1)	(2)	(3)	(4)	(5)	(6)
Decentralization of health services	0.2280**	0.2216**	−1.504**	−0.3606**	2.5583***	0.8949***
	(0.1149)	(0.1099)	(0.6823)	(0.1749)	(0.5704)	(0.3456)
Individual characteristics	Y	Y	Y	Y	Y	Y
Household characteristics	Y	Y	Y	Y	Y	Y
City characteristics	Y	Y	Y	Y	Y	Y
Individual fixed effects	Y	Y	Y	Y	Y	Y
Time fixed effect	Y	Y	Y	Y	Y	Y
N	93,603	93,603	19,609	19,609	37,013	37,013
Pseudo R^2^ / R^2^	0.1510	0.2195	0.0377	0.0380	0.3265	0.3445

Due to the lack of provincial-level health expenditure data in some regions and certain years, the observed values of the robustness test have decreased compared with the baseline regression.

### 3.6 Exclude cities under separate state planning and sub-provincial cities

Generally speaking, cities under separate state planning and sub-provincial cities enjoy more economic and social management authority than other cities, which may cause the behavioral patterns of the governments of these cities to be different from other cities. Therefore, the fiscal relationship between superiors and subordinates may also have large differences (49). To avoid the impact of this factor on the regression results, this paper conducts regression after excluding cities under separate state planning and sub-provincial cities. The regression results in Table 7 show that decentralization of health services at the municipal level is generally conducive to improving the health level of residents. Compared with the baseline regression results, except for the slight decrease in the regression coefficient of self-rated health, the significance and regression coefficients of BMI standards, depression status, phrase test and mathematics test all increased. The reason may be that self-rated health is a subjective evaluation index, so there is a certain measurement error. It can be seen that the regression results confirm the robustness of the baseline regression results.

**Table 7:** Robustness test—excluding cities under separate planning and sub-provincial cities.

Variables	Physical health		Depression status		Cognitive ability	
	Self-rated health	BMI standards	Summation method	Factor method	Self-rated health	BMI standards
	(1)	(2)	(3)	(4)	(5)	(6)
Decentralization of health services	0.0057**	0.0051**	−0.1036***	−0.0268***	0.0394***	0.0143**
	(0.0026)	(0.0022)	(0.0099)	(0.0026)	(0.0133)	(0.0070)
Individual characteristics	Y	Y	Y	Y	Y	Y
Household characteristics	Y	Y	Y	Y	Y	Y
City characteristics	Y	Y	Y	Y	Y	Y
Individual fixed effects	Y	Y	Y	Y	Y	Y
Time fixed effect	Y	Y	Y	Y	Y	Y
*N*	88,343	88,343	20,253	20,253	33,598	33,598
Pseudo R^2^ / R^2^	0.1552	0.1916	0.0460	0.0461	0.6227	0.7189

## 4 Discussion

The benchmark regression in this study confirms that municipal-level health service decentralization has a promoting effect on residents’ multi-dimensional health, including improving residents’ physical health, alleviating depression, and improving cognitive abilities. Meanwhile, the benchmark regression is repeated using methods such as lagged one period of explanatory variables, instrumental variable method, replacing the measurement method of health, changing the measurement method of health service decentralization, excluding cities under separate state planning and sub-provincial cities to verify this impact again. It is notable that there may be differences in health effects and policy effects under different groups and scenarios. In order to explain the research results more comprehensively and put forward targeted policy recommendations, this study conducts further discussions. Specifically, an in-depth comparative analysis is conducted on resident groups with different household registration types, marital status, behavioral habits, geographic locations, education levels and household income levels.

### 4.1 Association between decentralization of health services at the municipal level and health outcomes, by household registration type

Due to differences in the living environment and production methods between rural residents and urban residents, decentralization of health services at the municipal level may have heterogeneous impacts on the health of rural and urban residents. In order to examine this impact, this paper divides residents into rural residents and urban residents according to household registration type, and then conducts sub-sample regression. The regression results in Table 8 show that in terms of physical health, decentralization of health services at the municipal level has a significant positive impact on the self-rated health of rural residents, but has no significant impact on the self-rated health of urban residents; While decentralization of health services at the municipal level has a significant positive impact on the BMI standards of urban residents, but has no positive impact on the BMI standards of rural residents. The possible explanation is that prefecture-level cities’ financial investment in health has significantly improved the medical conditions in rural areas. Therefore, rural residents’ self-evaluated health is more optimistic than that of urban residents. Meanwhile, urban residents pay more attention to objective indicators such as physical health quality, therefore, the impact of the BMI standard is more significant. In terms of depression status, decentralization of health services at the municipal level can alleviate the depression of rural residents and urban residents, which is beneficial to the mental health of rural residents and urban residents. In terms of cognitive ability, decentralization of health services at the municipal level can improve phrase test scores of rural residents and math test scores of urban residents. The possible explanation is that there are differences in the human capital structure, knowledge structure, knowledge acquisition methods, and thinking methods between rural residents and urban residents, which causes rural residents to focus more on memory cognitive abilities, while urban residents develop more inferential cognitive abilities.

**Table 8:** Further analysis—rural household registration and urban household registration.

Variables	Physical health		Depression status		Cognitive ability	
	Self-rated health	BMI standards	Summation method	Factor method	Self-rated health	BMI standards
	(1)	(2)	(3)	(4)	(5)	(6)
Panel A: Rural household registration						
decentralization of health services	0.0087***	0.0021	−0.0408***	−0.0107***	0.0316***	0.0069
	(0.0021)	(0.0017)	(0.0064)	(0.0017)	(0.0086)	(0.0043)
N	74,208	74,208	19,685	19,685	29,803	29,803
Pseudo R^2^ / R^2^	0.1551	0.1834	0.0424	0.0427	0.6113	0.7171
Panel B: Urban household registration						
decentralization of health services	−0.0001	0.0069**	−0.0376***	−0.0098***	0.0142	0.0149*
	(0.0033)	(0.0028)	(0.0135)	(0.0035)	(0.0139)	(0.0078)
N	26,469	26,469	3,245	3,245	8,221	8,221
Pseudo R^2^ / R^2^	0.1531	0.2273	0.0218	0.0216	0.4860	0.6683
Individual characteristics	Y	Y	Y	Y	Y	Y
Household characteristics	Y	Y	Y	Y	Y	Y
City characteristics	Y	Y	Y	Y	Y	Y
Individual fixed effects	Y	Y	Y	Y	Y	Y
Time fixed effect	Y	Y	Y	Y	Y	Y

Overall, the decentralization of health services at the municipal level has a better effect on promoting the multi-dimensional health of rural residents than that of urban residents. Since rural residents have less disposable wealth, their personal medical and health investment is less than that of urban residents. The improvement of rural residents’ medical and health conditions relies more on government investment (50). The decentralization of health services at the municipal level means that municipal governments invest more financial funds in the medical and health field, which has greatly improved the medical and health conditions of rural residents, and in turn significantly improved the health of rural residents.

### 4.2 Association between decentralization of health services at the municipal level and health outcomes, by marital status

Marriage can bring financial and emotional support to individuals, which has been confirmed by most studies (51, 52). Considering the differences in the characteristics of different groups, the effect of decentralization of health services at the municipal level on improving residents’ health may be different between married people and unmarried people. To analyze this differential impact, this paper divides residents into married people and unmarried people according to their marital status, and then conducts sub-sample regression. Table 9 reports the results of this regression. Among them, decentralization of health services at the municipal level has a significant improvement effect on the self-rated health of both married and unmarried people, but this improvement effect is more obvious for unmarried people. However, when it comes to other health indicators, the exact opposite is true. Decentralization of health services at the municipal level has a significant improvement effect on BMI standards, depression status, phrase test and mathematics test scores of married people than that of unmarried people. That is, regardless of the regression coefficient or the significance level, the health effect is better for married people.

**Table 9:** Further analysis—married people and unmarried people.

Variables	Physical health		Depression status		Cognitive ability	
	Self-rated health	BMI standards	Summation method	Factor method	Self-rated health	BMI standards
	(1)	(2)	(3)	(4)	(5)	(6)
Panel A: Married						
Decentralization of health services	0.0046**	0.0030*	−0.0432***	−0.0113***	0.0273***	0.0109***
	(0.0020)	(0.0018)	(0.0065)	(0.0017)	(0.0086)	(0.0042)
*N*	73,361	73,361	18,428	18,428	29,658	29,658
Pseudo R^2^ / R^2^	0.1298	0.2703	0.0355	0.0356	0.5777	0.6930
Panel B: Unmarried						
Decentralization of health services	0.0108***	0.0026	−0.0302**	−0.0081**	0.0254*	−0.0011
	(0.0039)	(0.0026)	(0.0125)	(0.0032)	(0.0135)	(0.0081)
*N*	27,316	27,316	4,502	4,502	8,366	8,366
Pseudo R^2^ / R^2^	0.2189	0.0864	0.0404	0.0412	0.7208	0.7799
Individual characteristics	Y	Y	Y	Y	Y	Y
Household characteristics	Y	Y	Y	Y	Y	Y
City characteristics	Y	Y	Y	Y	Y	Y
Individual fixed effects	Y	Y	Y	Y	Y	Y
Time fixed effect	Y	Y	Y	Y	Y	Y

The possible explanation is that there is a certain degree of subjectivity in self-rated health, and the self-rated health status of unmarried people is more optimistic than that of married people. Meanwhile, married people can obtain emotional support from their partners, share social resources, and exert family economies of scale. This welfare effect can relieve individual stress, ensure individuals invest more in health care expenditures, and improve individual health. Overall, decentralization of health services at the municipal level has a more obvious health-promoting effect on married people.

In addition, there are differences in the sensitivities of sub-indicators. Specifically, for married people, the depression status (summation method), depression status (factor method), phrase test and mathematics test indicators are more sensitive than other indicators. This shows that the biggest benefit of marriage is that the emotional support it brings can relieve stress, and family and social activities can form cognitive stimulation. However, although married people may have regular living habits, they may also undertake more family responsibilities and pressure, such as housework and childcare, so the effect on physical health is not that obvious. For unmarried people, self-rated health indicator is more sensitive than other indicators. One of the possible explanations is that unmarried people do not need to undertake family responsibilities and pressure.

### 4.3 Association between decentralization of health services at the municipal level and health outcomes, by behavioral habits

Smoking behavior affects an individual’s physical fitness and emotions. Therefore, the effect of decentralization of health services at the municipal level on residents’ health may be different between smokers and non-smokers. To verify whether this difference exists, this paper divides individuals into smokers and non-smokers based on the behavior of smoking, and then performs sub-sample regression. The regression results in Table 10 show that decentralization of health services at the municipal level has a more prominent role in promoting the physical health and cognitive abilities of non-smokers, and the regression coefficient and significance level are better than that of smokers. Meanwhile, decentralization of health services at the municipal level has a significant alleviation effect on the depression of both smokers and non-smokers. However, decentralization of health services at the municipal level alleviates the depression of smokers to a greater extent than non-smokers.

**Table 10:** Further analysis—smoking and non-smoking groups.

Variables	Physical health		Depression status		Cognitive ability	
	Self-rated health	BMI standards	Summation method	Factor method	Self-rated health	BMI standards
	(1)	(2)	(3)	(4)	(5)	(6)
Panel A: Smoking						
decentralization of health services	0.0008	0.0019	−0.0657***	−0.0170***	0.0113	0.0117*
	(0.0034)	(0.0029)	(0.0106)	(0.0027)	(0.0140)	(0.0071)
*N*	27,054	27,054	6,976	6,976	10,858	10,858
Pseudo R^2^ / R^2^	0.1166	0.2450	0.0431	0.0436	0.5092	0.6525
Panel B: Non-smoking						
decentralization of health services	0 0.0068***	0.0013	−0.0285***	−0.0076***	0.0343***	0.0100**
	(0.0022)	(0.0018)	(0.0069)	(0.0018)	(0.0087)	(0.0045)
*N*	68,346	68,346	15,953	15,953	27,104	27,104
Pseudo R^2^ / R^2^	0.1666	0.2089	0.0407	0.0408	0.6538	0.7461
individual characteristics	Y	Y	Y	Y	Y	Y
Household characteristics	Y	Y	Y	Y	Y	Y
City characteristics	Y	Y	Y	Y	Y	Y
Individual fixed effects	Y	Y	Y	Y	Y	Y
Time fixed effect	Y	Y	Y	Y	Y	Y

Many studies have shown that smoking is addictive and a major cause of chronic diseases. Therefore, the physical functions, cognitive abilities and behavioral habits of non-smoking people are often better than those of smoking groups. The decentralization of health services at the municipal level allows municipal governments to have more funds and autonomy to carry out health education and health promotion activities. These activities can more easily encourage non-smoking people to form healthy lifestyles and behavioral habits. Therefore, decentralization of health services at the municipal level has a more significant improvement effect on physical health and cognitive abilities of non-smoking groups. In addition, nicotine component in tobacco can relieve anxiety, enhance the duration of positive emotions and self-perceived energy. Thus, smoking is regarded as a way to cope with psychological stress (53). Based on this, it is generally believed that people who smoke more frequently face more anxiety than those who smoke less frequently. The decentralization of health services at the municipal level allows municipal governments to provide psychological counseling services and social network support to residents, which has a more obvious effect on improving the mental health of smoking groups. Overall, decentralization of health services at the municipal level has a more obvious health-promoting effect on non-smoking groups.

### 4.4 Association between decentralization of health services at the municipal level and health outcomes, by geographic locations

Theoretically, there are differences in economic conditions, social environment, medical conditions, resource endowments and development strategies in different regions, and the effect of health decentralization may be affected. To further examine the regional heterogeneity of the impact of decentralization of health services at the municipal level on residents’ health, according to traditional classification method, this paper divides the total sample into three sub-samples: eastern region, central region and western region, and then conducted sub-sample regression. The regression results are shown in Table 11. In terms of depression status, decentralization of health services at the municipal level has significantly alleviated the depression status of people in the eastern, central and western regions. Among them, this mitigation effect has the most obvious impact in the western region, followed by central region, and has a weaker impact in the eastern region. This phenomenon is in line with expectations. Compared with eastern region, central and western regions have a slower pace of life and less work pressure. The mental health status of people in this region is generally good. Decentralization of health services at the municipal level has a more significant alleviating effect on depression among the population in this region.

**Table 11:** Further analysis—Eastern region, central region and western region.

Variables	Physical health		Depression status		Cognitive ability	
	Self-rated health	BMI standards	Summation method	Factor method	Self-rated health	BMI standards
	(1)	(2)	(3)	(4)	(5)	(6)
Panel A: Eastern region						
Decentralization of health services	0.0084***	0.0007	−0.0160*	−0.0042**	0.0356***	0.0039
	(0.0026)	(0.0021)	(0.0083)	(0.0021)	(0.0110)	(0.0056)
*N*	43,823	43,823	9,887	9,887	16,544	16,544
Pseudo R^2^ / R^2^	0.1615	0.1913	0.0486	0.0487	0.5912	0.7193
Panel B: Central region						
Decentralization of health services	−0.0178***	0.0032	−0.0394***	−0.0103***	0.0120	0.0254***
	(0.0033)	(0.0027)	(0.0119)	(0.0031)	(0.0135)	(0.0070)
*N*	40,123	40,123	8,700	8,700	14,582	14,582
Pseudo R^2^ / R^2^	0.1539	0.1933	0.0327	0.0332	0.6287	0.7247
Panel C: Western region						
Decentralization of health services	0.0124**	−0.0047	−0.1097***	−0.0287***	−0.0546**	−0.0187
	(0.0054)	(0.0054)	(0.0221)	(0.0057)	(0.0260)	(0.0144)
*N*	16,731	16,731	4,343	4,343	6,898	6,898
Pseudo R^2^ / R^2^	0.1502	0.2069	0.0954	0.0950	0.6453	0.7191
Individual characteristics	Y	Y	Y	Y	Y	Y
Household characteristics	Y	Y	Y	Y	Y	Y
City characteristics	Y	Y	Y	Y	Y	Y
Individual fixed effects	Y	Y	Y	Y	Y	Y
Time fixed effect	Y	Y	Y	Y	Y	Y

There are 10 in the eastern region, namely Beijing, Tianjin, Hebei, Liaoning, Shanghai, Jiangsu, Zhejiang, Fujian, Shandong, and Guangdong; and 8 in the central region, namely Shanxi, Jilin, Heilongjiang, Anhui, Jiangxi, Henan, Hubei, Hunan; 7 in the western region, namely Sichuan, Chongqing, Guizhou, Yunnan, Shaanxi, Gansu, and Guangxi.

Different from depression status, the impact of decentralization of health services at the municipal level on residents’ physical health and cognitive ability shows different characteristics in the eastern, central and western regions. Among them, in terms of physical health, decentralization of health services at the municipal level has improved the health of people in eastern and western regions, but reduced the health of people in central region. In terms of cognitive ability, decentralization of health services at the municipal level improves the cognitive ability of people in eastern and central regions, but reduces the cognitive ability of people in western region. The possible explanation is that decentralization of health services weakens the redistribution ability of higher-level governments but emphasizes the initiative of local governments in managing medical and health affairs. However, compared with eastern region, the governance capacity of local governments in the central and western regions is relatively weak. There is more administrative paperwork after decentralization than under centralization, and redundant paperwork may result in duplication of medical services. Meanwhile, decentralization may cause local decision-makers to be reluctant to exercise their authority, which may lead to inefficient location selection of medical institutions and inefficient pricing of inputs (23). In addition, the central and western regions have relatively weak financial security capabilities, and the decentralization of health services has increased the financial pressure on local governments. Local governments, especially those in poor areas with insufficient income, may withdraw from investment projects in the medical and health field to a large extent (29). These factors have led to the relative inefficiency of medical and health expenditures of local governments in the central and western regions. As a result, the physical health and cognitive abilities of residents in these regions have not been uniformly improved. Overall, the above results verify that decentralization of health services at the municipal level will expand inter-regional health inequalities.

### 4.5 Association between decentralization of health services at the municipal level and health outcomes, by educational level

According to the different education levels of individuals, this paper divides the samples into primary school and below, junior high school, high school, college, and university degree and above groups, then conducts sub-sample regression. The regression results are shown in Table 12. For individuals with primary school education and below, decentralization of health services at the municipal level has significantly improved the individual’s self-rated health status, reduced depression, and improved memory cognitive abilities. For individuals with junior high school education, decentralization of health services at the municipal level also has a similar impact. What is slightly different is that the health improvement effect of individuals with primary school education and below is more obvious than that of individuals with junior high school education. It is notable that the health improvement effect of decentralization of health services at the municipal level on individuals with higher educational levels is not obvious. Specifically, for individuals with high school education, decentralization of health services at the municipal level increases the individual’s BMI standard and reduces the degree of depression. For individuals with college education, decentralization of health services at the municipal level has no significant impact on individual health. For individuals with university degree and above, decentralization of health services at the municipal level reduces individuals’ self-rated health and increases BMI standard.

**Table 12:** Further analysis—primary school and below, junior high school, high school, college, and university degree and above.

Variables	Physical health		Depression status		Cognitive ability	
	Self-rated health	BMI standards	Summation method	Factor method	Self-rated health	BMI standards
	(1)	(2)	(3)	(4)	(5)	(6)
Primary school and below	0.0066***	−0.0025	−0.0504***	−0.0133***	0.0410***	−0.0072
	(0.0024)	(0.0023)	(0.0095)	(0.0025)	(0.0122)	(0.0052)
Junior high school	0.0062*	0.0035	−0.0441***	−0.0115***	0.0318***	0.0041
	(0.0033)	(0.0026)	(0.0093)	(0.0024)	(0.0123)	(0.0058)
High school	0.0082	0 0.0065*	−0.0221*	−0.0056	0.0019	0.0088
	(0.0051)	(0.0038)	(0.0133)	(0.0034)	(0.0137)	(0.0094)
College	0.0004	0.0021	−0.0230	−0.0061	−0.0162	0.0141
	(0.0093)	(0.0063)	(0.0212)	(0.0054)	(0.0211)	(0.0141)
University degree and above	−0.0247**	0.0184**	−0.0265	−0.0068	−0.0033	−0.0219
	(0.0123)	(0.0080)	(0.0293)	(0.0076)	(0.0230)	(0.0184)

Since table involves 30 regression equations, and due to space limitations, only estimated coefficients of key variables are listed.

Taken together, whether in terms of physical health, depression, or cognitive abilities, the decentralization of health services at the municipal level has had a more significant improvement effect on individuals with lower levels of education. The main possible reasons for the above phenomenon are: At present, the uneven distribution of medical resources still exists, and the health literacy and cognitive abilities of individuals vary depending on their education level. Individuals with higher education levels tend to have stronger access to medical and health resources, as well as higher levels of health knowledge and cognitive abilities. With the implementation and advancement of decentralization of health services at the municipal level, the service capacity and efficiency of medical resources have been improved. For individuals with lower education levels, the decentralization of health services at the municipal level has greatly increased the availability of medical resources and significantly enhanced their own medical and health knowledge reserves. However, the above-mentioned marginal impact of municipal-level health service decentralization on individuals with higher education levels is relatively small. Therefore, decentralization of health services at the municipal level has a more significant improvement effect on physical health, depression status, and cognitive abilities among individuals with lower education levels.

### 4.6 Association between decentralization of health services at the municipal level and health outcomes, by household income level

This paper divides the sample into low-income, lower-middle income, middle-income, upper-middle income and high-income groups based on *per capita* household income. Among them, individuals in the top 20% of *per capita* income are low-income groups, individuals in the 20–40% are middle-income group, individuals in the 40–60% are middle-income group, and individuals in the 60–80% are upper-middle income group, and individuals between 80 and 100% are high-income group. This paper conducts sub-sample regression, and regression results are shown in Table 13. It is notable that decentralization of health services at the municipal level has a greater effect on improving the physical health of individuals with higher incomes, a stronger effect on improving the depression status of individuals with lower incomes, and a more significant effect on improving the cognitive ability of lower-middle and upper-middle income individuals.

**Table 13:** Further analysis—low, lower-middle, middle, upper-middle and high income groups.

Variables	Physical health		Depression status		Cognitive ability	
	Self-rated health	BMI standards	Summation method	Factor method	Phrase test	Mathematics test
	(1)	(2)	(3)	(4)	(5)	(6)
Low	0.0050	−0.0041	−0.0544***	−0.0143***	0.0040	−0.0052
	(0.0047)	(0.0040)	(0.0148)	(0.0038)	(0.0210)	(0.0106)
Lower-middle	0.0011	−0.0012	−0.0508***	−0.0133***	0 0.0325**	0.0143*
	(0.0042)	(0.0035)	(0.0108)	(0.0028)	(0.0165)	(0.0083)
Middle	0.0018	0.0021	−0.0624***	−0.0163***	−0.0110	−0.0069
	(0.0038)	(0.0031)	(0.0107)	(0.0028)	(0.0160)	(0.0081)
Upper-middle	0.0110***	0.0056*	−0.0058	−0.0014	0.0510***	0.0270***
	(0.0035)	(0.0029)	(0.0119)	(0.0031)	(0.0151)	(0.0078)
High	−0.0015	0.0090***	0.0037	0.0009	0.0185	0.0059
	(0.0040)	(0.0034)	(0.0165)	(0.0042)	(0.0156)	(0.0085)

Since table involves 30 regression equations, and due to space limitations, only estimated coefficients of key variables are listed.

The main possible reasons for the above phenomenon are: In terms of physical health, the medical and health services available to individuals are not only affected by the distribution of medical resources, but also vary according to income levels. Individuals with higher incomes have relatively strong affordability for medical health expenses, and relatively abundant funds for obtaining medical resources for physical health. Decentralization of health services at the municipal level may enable local governments to respond more flexibly to the needs of local residents, including the needs of high-income groups for high-quality medical services. In turn, high-income groups may have easier access to high-quality medical services, and the positive impact of health decentralization on their physical health is more pronounced. In terms of depression status, individuals with lower incomes have relatively weak awareness of mental health problems and have relatively few funds available for the treatment of mental illnesses, which requires the intervention of local health agencies and health financial investment from local governments. Decentralization of health services at the municipal level is conducive to increasing the intervention intensity of local health institutions in residents’ psychological problems and the level of local financial investment in health, which in turn significantly alleviates the depression status of individuals with lower incomes. In terms of cognitive ability, decentralization of health services at the municipal level has a significant positive impact on phrase test and mathematics test scores of lower-middle income and upper-middle income groups. The possible explanation is that the working hours and intensity of lower-middle and upper-middle income groups are higher than those of other groups, and their working methods enable these groups to develop memory and inferential cognitive abilities. Therefore, decentralization of health services at the municipal level has a more obvious promotion effect on the cognitive abilities of lower-middle and upper-middle income groups.

### 4.7 Limitations

The following points can be made as limitations of this research, which offer a wide scope.

for future studies. First, CFPS data is obtained based on surveys. Different survey methods may have a certain impact on data quality, such as face-to-face interviews, telephone interviews, etc. Second, there is relatively insufficient macro data on China’s medical and health conditions, and therefore there is a lack of objective indicators for measurement. This also leads to the lack of analysis of the intermediary effect in this study. In the future, after further collecting or obtaining relevant data, the analysis of mechanism effects can be enriched. Third, the research in this study is based on the individual level. In the future, analysis can be carried out on the family level to discuss the impact of decentralization of health services at the municipal level on family economy, commercial insurance purchase decisions, etc. Fourth, we can then select studies from other years to discuss the impact of decentralization of health services at the municipal level on residents’ multi-dimensional health during the COVID-19 epidemic, compare the differences in regression results in different periods, and further enrich research on related topics in this field.

## 5 Conclusion

National health is the primary goal of medical development and the basic condition for sustainable economic and social development. Reasonable allocation relationship of intergovernmental health expenditure can help play a positive role in effective government and achieve effective connection with the goal of public health. Therefore, this study takes decentralization of health services at the municipal level as the research object and explores its health effects, which not only enriches the research on fiscal decentralization theory, but also enriches the research on global health interventions in resource-limited settings.

Based on the perspective of multi-dimensional health, from the three aspects of physical health, depression status and cognitive ability, the six sub-indicators of self-rated health, BMI standards, depression scores (summation method), depression scores (factor method), phrase test scores and mathematics test scores are discussed separately. The results show that the decentralization of health services at the municipal level has a significant promotion effect on the multi-dimensional health of residents. Afterwards, this paper conducts a series of robustness tests and found that the above conclusions still hold. Further analysis shows that: (a) For rural residents, decentralization of health services at the municipal level helps improve self-evaluated health, mental health, and memory cognitive abilities; for urban residents, decentralization of health services at the municipal level helps improve BMI standards, mental health and calculated cognitive abilities. (b) Decentralization of health services at the municipal level generally has a more obvious health-promoting effect on married group, non-smoking group, eastern region group, and groups with lower educational levels. (c) Decentralization of health services at the municipal level has significant improvement effect on the mental health of lower income groups, the physical health of higher income groups, and the cognitive ability of lower-middle and upper-middle income groups.

This study provides the following implications for policy implementation and design in China: (a) In the implementation of decentralization policies, the administration rights and expenditure responsibilities of higher-level governments should be appropriately strengthened, so as to avoid inter-regional inequalities in medical conditions and residents’ health. (b) Further clarify the health expenditure responsibility sharing mechanism among governments below the provincial level to build a clear and efficient medical and health system. (c) Under the fiscal decentralization system, lower-level governments assume the main expenditure responsibilities. To reduce the financial burden on lower-level governments, taxes on addictive consumer goods such as tobacco and alcohol can be earmarked for medical and health expenditures of governments, or social capital can be introduced to provide some medical products. (d) Since some health matters have obvious externalities, the scope of benefits may exceed the jurisdictional boundaries of local governments. In this case, building a cross-regional flexible joint prevention mechanism can help medical services cover more people. (e) The research shows that decentralization of health services at the municipal level can play an important role in improving public health. However, sub-sample regression results show that there are large differences in health-promoting effects of decentralization of health services at the municipal level on different regions and groups. To avoid the exacerbation of health inequalities between groups and regions with different characteristics, this requires governments at different levels to dynamically adjust when determining the proportion of their fiscal health expenditures. Through the dynamic adjustment mechanism, the staged development goals of medical and health services can be achieved, and the physical health, mental health and cognitive abilities of residents can also be greatly improved.

## Data availability statement

The original contributions presented in the study are included in the article/supplementary material, further inquiries can be directed to the corresponding author.

## Author contributions

FC: Conceptualization, Formal analysis, Project administration, Resources, Software, Visualization, Writing – original draft, Writing – review & editing. SW: Funding acquisition, Investigation, Methodology, Software, Supervision, Validation, Writing – original draft, Writing – review & editing.
